# Gender differences in depression, anxiety, and quality of life in Parkinson’s disease before and after deep brain stimulation surgery: a multicentre cohort study

**DOI:** 10.1136/bmjno-2025-001246

**Published:** 2025-09-30

**Authors:** Molly G Abbott, Arteen Ahmed, Nicola Pavese, Antonella Macerollo, Edward J Newman, Jibril Osman Farah, Nagaraja Sarangmat, Anjum Misbahuddin, David Ledingham, Michelle GIbbs, Russell Mills, Keyoumars Ashkan, Monty Silverdale, Michael Samuel, David Okai, Paul Shotbolt

**Affiliations:** 1 King’s College London Institute of Psychiatry Psychology & Neuroscience, London, UK; 2 Newcastle Upon Tyne Hospitals NHS Foundation Trust, Newcastle upon Tyne, UK; 3 The Walton Centre NHS Foundation Trust, Liverpool, UK; 4 Neurology, Queen Elizabeth University Hospital, Glasgow, UK; 5 Oxford University Hospitals NHS Foundation Trust, Oxford, UK; 6 Barking Havering and Redbridge University Hospitals NHS Trust, Romford, UK; 7 Salford Royal NHS Trust, Salford, UK

**Keywords:** DEPRESSION, PARKINSON'S DISEASE, NEUROSURGERY

## Abstract

**Background:**

Disproportionately fewer females with Parkinson’s disease (PD) undergo deep brain stimulation surgery (DBS). Some data show worse depression, anxiety, and quality of life (QOL) in females with PD. Investigations into these gender disparities, or the effect of DBS on these non-motor symptoms, remain limited.

**Methods:**

61 PD patients across seven UK DBS centres were recruited for the Clinical Response of Impulsive behaviours to deep brain Stimulation in PD (CRISP) prospective cohort study. Questionnaires measured primary outcomes of depression (Patient Health Questionnaire-9), anxiety (Generalised Anxiety Disorder-7) and QOL (Parkinson’s Disease Questionnaire-39) before and 6 months after bilateral subthalamic nucleus DBS, and secondary outcomes of predictors of postoperative changes in mood.

**Results:**

Females were disproportionately under-referred for DBS (28% of cohort). Baseline depression and anxiety were similar between genders. While DBS significantly improved overall anxiety (p<0.001), females reported significantly more postoperative anxiety than males (median score 7 vs 1.5*,* Cohen’s *d*=0.33, p=0.009). Postoperatively, only males experienced a significant reduction in moderate depression, by 29% (p=0.004) (12% in females). QOL improved significantly by similar proportions, thus significantly worse QOL in females preoperatively was sustained as 9.12% worse postoperatively (Cohen’s d*=*0.75, p=0.02). Preoperatively, females reported significantly worse mobility, social support, and pain; postoperatively, the significant difference in mobility was sustained. Longer PD duration, worse QOL, and mobility predicted postoperative depression (R^2^*=*0.156*,* p=0.003), while female gender and reduced social support predicted postoperative anxiety (R^2^=0.23, p<0.001).

**Conclusions:**

DBS showed clinical efficacy for non-motor PD symptoms across genders, evidencing the need to close the gender gap in DBS. Analysis by gender highlighted significant disparities and postoperative predictors that provide impetus for tailored DBS counselling.

**WHAT IS ALREADY KNOWN ON THIS TOPIC**

- There are sparse studies that investigate the reasons for fewer females undergoing deep brain stimulation surgery (DBS), with proposed reasons including reduced patient preference, increased depression, and reduced clinician referrals.
- Anxiety and depression affect 40%–50% of patients with Parkinson’s disease (PD), yet the gender-differentiated effect of DBS on these non-motor PD symptoms is underexplored and pertinent to inform DBS counselling clinics.

**WHAT THIS STUDY ADDS**

- Together these data add novel characterisation of gender differences in a DBS cohort. Contrasting to previous data, we identify a similar prevalence of preoperative depression and anxiety, and find predictors of improved depression and anxiety at 6-month postoperatively.
- Our study adds to evidence that there is a disproportionately reduced proportion of females in DBS cohorts. Females experience significantly worse preoperative quality of life (QOL); with worse mobility, pain, social support, and carer engagement, which may contribute to females’ known reduced personal preference for DBS.
- We show DBS to be clinically efficacious for both genders, with significant improvements in QoL and anxiety, however a significant improvement in clinical (moderate) depression in males only.

**HOW THIS STUDY MIGHT AFFECT RESEARCH, PRACTICE OR POLICY**

- Preoperative DBS clinics encounter patient questions on how DBS may impact their depression, anxiety, and QOL, which this study helps to inform. This evidence supports the practice of tailored DBS counselling, particularly with consideration of reduced social support in females preoperatively.
- More research is required to understand why there is a gender difference in the motor and non-motor symptoms of PD.
- Our study provides impetus for investigation into the barriers to recruitment of females and ethnic minorities in DBS cohorts, to inform healthcare strategies that can address inequalities in functional neurosurgery cohorts.

## Introduction

 Parkinson’s disease (PD) is the second most common chronic neurodegenerative disorder, causing progressive motor and non-motor symptoms.^1^ Deep brain stimulation surgery (DBS) is a routine National Health Service (NHS)-funded treatment for PD motor symptoms.^2^ However, its effect on non-motor symptoms, particularly depression and anxiety, which affect 40%–50% of PD patients^3^ and correlate with poorer prognosis^4^ and postoperative quality of life (QOL),^1^ is less well characterised.

Research on gender differences in PD symptoms is limited, however, findings indicate differences in preoperative and postoperative motor symptoms,^5^ including later development of motor symptoms and increased dyskinesias in females.^6^ Non-motor symptoms seem to also vary by gender, with females reporting more depression^6^ and anxiety,^7^ though studies remain heterogeneous, limited for DBS cohorts, and have not characterised the effect of DBS on non-motor symptoms by gender.

Females are disproportionately under-represented within DBS cohorts, comprising only 32%,^8^ despite making up 40% of the general PD population.^9^ This disparity remains a challenge to understand since there are few focused studies on gender differences in PD.^5^ Females report challenges from their caregiver roles,^10^ more frequently living alone,^11^ reduced healthcare-seeking behaviours and personal preference for DBS,^6 12^ and greater rejection for DBS due to depression severity.^6^ Females report poorer disease-related QOL,^1^ and one study found greater long-term improvement in QOL after DBS for males despite equal motor improvement,^13^ which could be attributable to an unequal non-motor symptom improvement.

### Rationale and objectives

We conducted a novel analysis of gender differences in clinical response to DBS for anxiety, depression, and QOL in PD, to address current knowledge gaps and explore reasons for fewer females accessing DBS. We hypothesise that females experience less social support and more social isolation, contributing to reduced healthcare-seeking behaviours and personal preference for DBS.^6 12^

We utilised data from seven DBS centres within the UK National DBS Network, ensuring findings are generalisable across the PD population. The outcomes of this study inform healthcare professionals of gender differences that may be barriers to accessing DBS and experiencing improved QOL postoperatively. We aim to provide data to inform tailored preoperative and postoperative counselling.

## Methods

This observational cohort study analyses data from the Clinical Response of Impulsive behaviours to deep brain Stimulation in PD (CRISP) study. A project grant was awarded by the charity Parkinson’s UK (H-2303). The CRISP study protocol was reviewed and advised on by several groups, including lay and expert groups of PD patients and carers and the charity Parkinson’s UK, for patient and public involvement.

### Participant recruitment

All centres within the UK National DBS Network were invited to participate in the CRISP study, of which seven agreed to be involved (online supplemental table 1). The CRISP study recruitment started in December 2021, and the dataset for this cohort study was archived on 3 September 2024. The local investigators at each centre recruited potential participants by verbal invitation. Participants who agreed were sent introductory packages that included informed consent forms for enrolment and baseline questionnaires.

Participants were recruited according to predefined eligibility criteria:

Inclusion criteria

- Patients referred for DBS as part of standard care for motor symptoms of PD.
- Patients deemed eligible after neuropsychiatric screening assessment.
- Fluent in English language.

Exclusion criteria

- Nil.

### Data collection

All participants underwent bilateral subthalamic nucleus DBS. The CRISP study collected data prospectively at 3, 6, and 12 months. The dataset collected at the 6-month timepoint was selected for this cohort study to allow time for adjustment since DBS. At the time of analysis and writing, 12-month data collection was incomplete and ongoing. Therefore, this study used two data points from before DBS (preoperative baseline, T0) and 6 months after DBS (postoperative, T2). The only missing data were part III baseline scores for the Movement Disorder Society-sponsored revision of the Unified Parkinson’s Disease Rating Scale (MDS-UPDRS),^14^ with only n=13 reported. The study size was arrived at by using all data collected by the CRISP study at T0 and T2. Data collection in the CRISP study was managed by the local investigators at each research centre. All data were saved, collated, and anonymised by the lead research fellow before distribution.

The following questionnaires were used in this analysis:

#### Generalised Anxiety Disorder-7

A seven-item, self-report questionnaire that scores generalised anxiety disorder (GAD) symptoms out of 20. Each item is scored from 0 to 3, with a total score of 5 denoting mild anxiety; 10, moderate anxiety; and 15, severe anxiety.^15^ A score of 10 or greater was the cut-off used to define a case of GAD, as per a previous study identifying this as a valid diagnostic criterion with >80% sensitivity and specificity, when compared with independent diagnoses made by mental health professionals.^15^ We could not identify any literature that specifically investigated the most suitable anxiety screening questionnaire or cut-offs within a PD population.

#### Patient Health Questionnaire-9 items

A nine-item, self-report questionnaire that scores the severity of depression over time.^16^ Each item is scored from 0 to 3, with a total score of 5 denoting mild depressive symptoms; 10, moderate; 15, moderately severe and 20, severe depressive symptoms.^17^ A sensitive cut-off score specific to a PD population has been identified previously as 9 or greater for clinically significant depression,^17^ hence this cut-off was used. The PHQ-9 was chosen for the CRISP study as it includes questions on somatic and pain-related symptoms that PD patients more frequently experience than the general population with depression, and it is shown to identify changes over time.^18^

#### Parkinson’s Disease Questionnaires—39 items

A self-report questionnaire with 8 domains of QOL specific to PD, to measure the impact of disease and treatments including DBS.^19^ The Parkinson’s Disease Summary Index (PDSI) is a summary score of health-related QOL using all eight domains (calculated using total PDQ-39-dimension scores divided by 8), with a maximum score of 100 that can be expressed as a percentage; 100% representing most severe impact of PD on QOL.^19^ We also report individual scores of the 7 domains of mobility, activities of daily living (ADL), social support, stigma, cognition, communication, and pain. We did not report the eighth domain, emotional well-being, given this aspect is more thoroughly reported by the GAD-7 and PHQ-9 scores.

### Statistical analysis

Preoperative and postoperative data variables were analysed using SPSS V.29.0.2.0 software (online supplemental table 2). The distributions of all datasets (cohort and categorised by gender) were assessed for normality by Shapiro-Wilk test (p<0.05 to reject null hypothesis of normality) and histogram visualisation. For all tests, exact two-tailed p values were used with an alpha-level of 0.05.

The following statistical analyses were performed:

1. *Comparison of cohort difference in PDQ-39 (PDSI and domains), GAD-7, and PHQ-9, from baseline to postoperative scores*.

The change in paired scores from baseline (T0) to 6 months postoperative (T2) was analysed using paired samples t-test, or Wilcoxon matched-pair sign rank test (for social support and pain). Difference scores were calculated as: (T0–T2) for GAD-7, PHQ-9 and PDQ-39 Summary index; and (T2–T0) for all PDQ-39 domains.

1. *Comparison of gender difference in PDQ-39 (PDSI and domains), GAD-7 and PHQ-9, at baseline, change after DBS, and post-operative scores*.

Male and female scores were compared using independent samples t-test, or independent samples Mann-Whitney U test. The following datasets split by gender were non-normally distributed: baseline values for PHQ-9, GAD-7, social support, stigma, and communication; difference (T0–T2) values for GAD-7; and postoperative values for GAD-7, PHQ-9, social support, stigma, mobility, communication, ADL. Presence of a clinical case of depression and anxiety was defined as those reaching the defined cut-off scores, and proportions were compared using McNemar’s χ^2^ test. Gender differences in mean doses of PD medications and levodopa equivalent daily dose (LEDD) were calculated using independent t-tests at baseline, difference scores (T0–T2), and 6 months postoperative (online supplemental table 3). Overall cohort reduction was calculated using paired t-tests. Effect sizes were calculated for t-tests using Cohen’s d with Hedges’ correction, and for Wilcoxon and Mann-Whitney U-tests using the *r* value equation (*r*=Z/√N).

1. *Analysis of the relationship between gender and QOL as predictors of the following dependent variables: change in PHQ-9, change in GAD-7 scores after DBS (T0–T2). All models were run separately for each dependent variable and included gender, PD duration, and the following other independent variables:*
  - *Model 1:* postoperative PDQ-39 domain scores (T2) to explore if any specific aspects of QOL correlate with postoperative depression or anxiety for each gender.
  - *Model 2:* baseline PDQ-39 domain scores (T0) to explore preoperative predictors for effect of DBS on depression and anxiety.
  - *Model 3*: baseline and postoperative PDSI scores to explore if overall QOL and/or gender are predictors of anxiety and depression.

Multivariate linear regression models were run for the above. Models 1 and 2 were exploratory models, using forward selection to identify covariates that were significant and did not reduce the model significance (calculated using Analysis of Variance (ANOVA)). Gender was coded such that a positive beta indicates correlation with female gender (male=0, female=1).

Note: the use of the term ‘gender’ throughout refers to the current gender orientation and biological sex assigned at birth for all participants.

## Results

**Figure 1:**
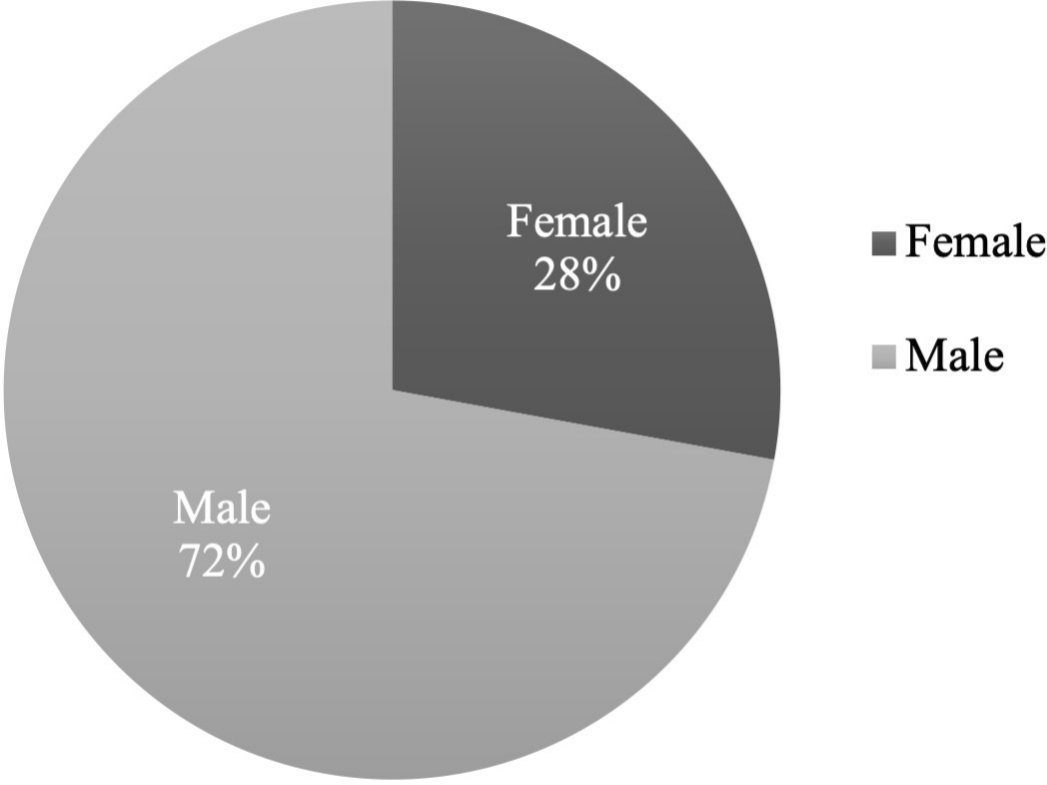
Females were underrepresented within our cohort of 61 patients with Parkinson’s disease undergoing deep brain stimulation surgery.

66 patients were referred and approved for DBS at the seven centres, which recruited a total of 61 participants, since 4 refused to join and 1 declined surgery. The surgery cohort consisted of 28% females (n=17) and 72% males (n=44) (figure 1). The ethnicity of participants was 96.7% white British, 1.6% Indian British, and 1.6% Arab. The average age at time of operation was 62.4 years (range 42–76) and mean duration of PD was 9.8 years (range 3–20). Baseline disease severity using mean MDS-UPDRS scores was as follows: part I=15.84 (SD=6.18), part II=20.3 (SD=7.82), part IV=11.62 (SD=3.98). Data for part III were provided for 13 participants: off-medication=48.2 (SD=9), on-medication=24 (SD=6.36).

### The effect of DBS on the whole cohort

The difference in baseline to postoperative scores showed improvement in QOL and clinical cases of anxiety and depression for the whole cohort. GAD-7 scores improved significantly by a mean decrease of 2.28 (p<0.01), and PHQ-9 scores improved, although this did not reach statistical significance (p=0.13) (online supplemental table 4). The proportion of clinical anxiety cases reduced from 18% to 8.2% (p=0.07), and clinical depression cases reduced significantly from 59% to 34% (p=0.003). QOL (PDSI) improved significantly by 8.32% overall (p<0.001) and improved within all PHQ-39 domains; with a significant reduction in disease impact on ADL by 9%, stigma by 8.69%, pain, and mobility by 6.73% (online supplemental table 4). PD medications use significantly reduced for LEDD (p≤0.001, Cohen’s *d=*0.736), and Levodopa, dopamine agonists and COMT (catechol-O-methyltransferase)-inhibitors (online supplemental table 3).

### Gender differences at baseline

The mean age of PD diagnosis was equal across genders (average 52.02 years in males, 52.18 years in females). Average PD duration in years was 11.2 (±4.84) for females and 9.2 (±3.43) for males. There was no significant gender difference in baseline MDS-UPDRS scores (online supplemental table 5).

**Figure 2:**
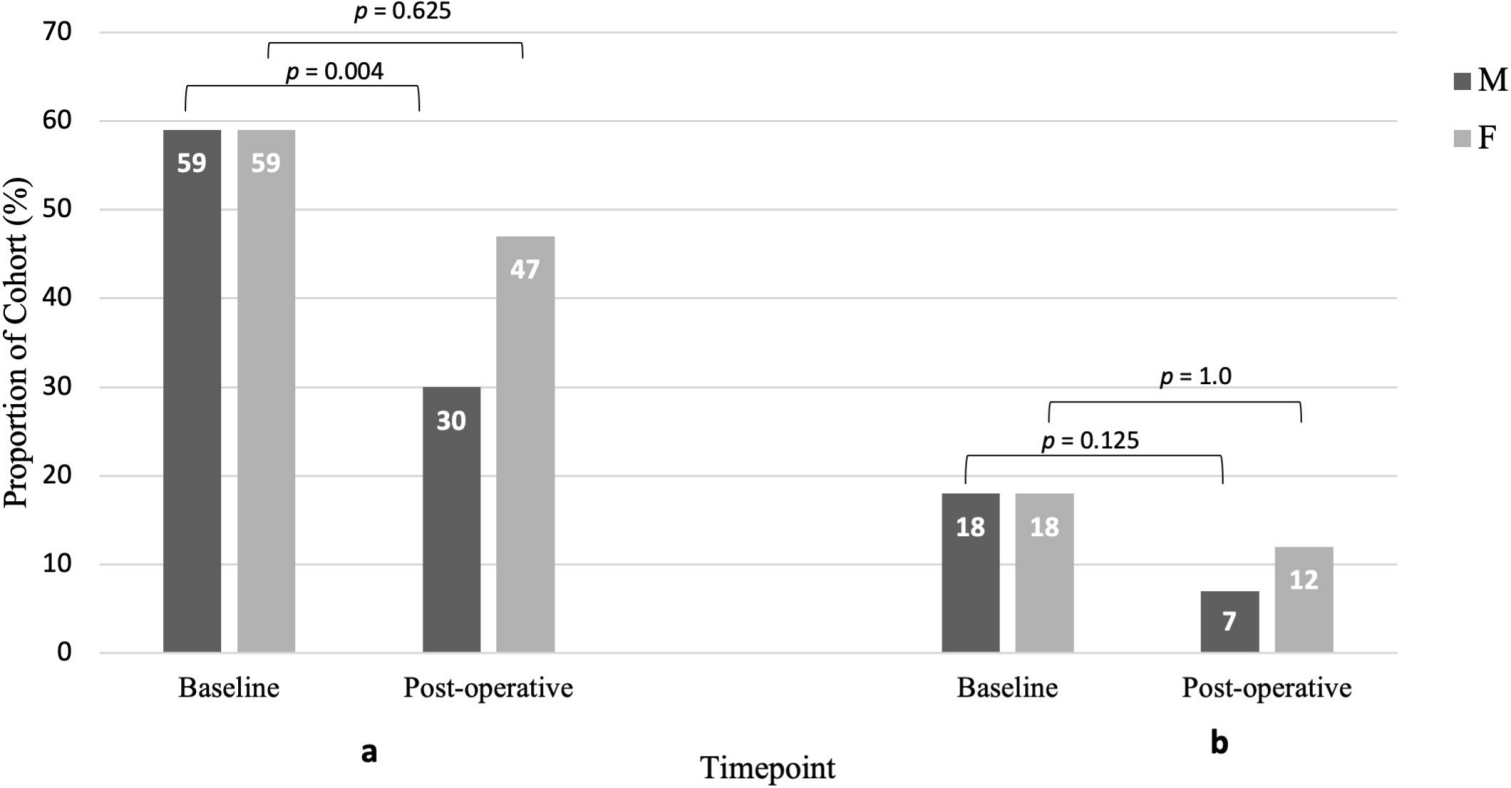
The proportion within males (M) and females (F) that reached cut-off for clinical depression (**a**) or anxiety (**b**) on questionnaire scores was equal at baseline, compared with 6 months postoperatively with only a significant reduction in cases in males. A GAD-7 cut-off score of 10 or greater was used to define a case of Generalised Anxiety Disorder. A PHQ-9 cut-off score of 9 or greater was used, representing moderate, clinically significant depression. Analysed using McNemar’s test χ^2^ test, reporting two-sided p values. GAD-7, Generalised Anxiety Disorder-7 questionnaire; PHQ-9, Patients' Health Questionnaire-9 items questionnaire.

At baseline, there was no significant difference in PHQ-9 or GAD-7 scores (table 1), though mean GAD-7 scores trended to be higher in females. In both genders, the proportion of cases of clinical depression and GAD that reached cut-off preoperatively was equal (figure 2). Overall QOL (PDSI) was significantly worse for females by 10.93% (SED 4.29, p=0.013, Cohen’s *d*=0.77). Females reported worse QOL scores in all individual domains; with significantly worse reported mean scores for pain (p=0.009, Cohen’s *d*=0.77), mobility (p=0.002, Cohen’s *d*=0.94), and social support (p=0.03, *r-value*=0.28) (table 1). Carer engagement was substantially reduced in females compared with males at baseline (11% and 29% respectively) and postoperatively (18% and 53% respectively). There was no gender difference in LEDD (p*=*0.433, Cohen’s *d=0.223*), nor in dose of PD medication types except for COMT inhibitors, which was significantly greater in males (p*=*0.02) (online supplemental table 3).

**Table 1:** Gender differences in quality of life, depression, and anxiety before deep brain stimulation surgery (DBS)

(a) Independent samples t-test							
Variable (T0)	Mean difference (SED)	Median* T0 (IQR)		Mean T0 (SD)		Effect size (Cohen’s d)	P value†
		M	F	M	F		
PDSI	10.93 (4.29)	N/A		32.98 (15.46)	43.91 (23.59)	0.77	**0.013**
Cognition	0.29 (0.87)	N/A		5.00 (3.17)	5.29 (2.71)	0.10	0.737
Pain	2.07 (0.76)	N/A		4.93 (2.61)	7.00 (2.81)	0.77	**0.009**
ADL	1.70 (1.58)	10.07 (5.52)	11.76 (5.61)	10.07 (5.52)	11.76 (5.61)	0.30	0.288
Mobility	10.93 (4.07)	16.20 (9.28)	24.76 (8.31)	16.20 (9.28)	24.76 (8.31)	0.94	**0.011**

(b) Independent samples Mann-Whitney U test						
Variable (T0)	U-value	Median T0 (IQR)		Z-test	Effect size (r value)	P value†
		M	F			
GAD-7	285.5	4.5 (7)	6 (6)	−1.429	−0.18	0.153
PHQ-9	364.5	10 (9)	9 (9)	−0.153	−0.02	0.878
Stigma	293	4 (7)	6 (7)	−1.309	−0.17	0.191
Social support	242.5	1 (2)	3 (5)	−2.164	−0.28	**0.03**
Communication	281.5	3 (3)	4 (5.5)	−1.505	−0.19	0.132

Pre-operatively, females reported worse overall quality of life (PDSI) and within the specific QOL domains of pain, mobility and social support.

* Median T0 values are provided for (a) to allow for direct comparison to data in (b) which were non-normally distributed.

† Two-sided p value, alpha level=0.05 (values reaching alpha level in bold face).

ADL, activities of daily living; F, female; GAD-7, Generalised Anxiety Disorder Questionnaire; M, male; PDQ-39, Parkinson’s Disease Questionnaire; PDSI, PDQ-39 Summary Index (overall score measure); PHQ-9, Patient Health Questionnaire for depression; T0, pre-operative baseline.

### Gender differences in the effect of DBS

A significant reduction in clinical cases of moderate depression was seen in males by 29% (p=0.004), however, not in females (figure 2). There was no statistically significant gender difference in the change in PHQ-9 scores (online supplemental table 6), or postoperative scores (table 2). Clinical anxiety cases were reduced by 11.2% (p=0.125) in males, and 6.2% in females (p=1.0) (figure 2). The gender difference in the postoperative reduction in GAD-7 scores was non-significant, though postoperative GAD-7 scores were significantly higher in females (median of 7 compared with 1.5 in males (p=0.009, Cohen’s d=0.33)) (table 2).

**Table 2:** Gender differences in anxiety, depression and quality of life scores at 6 months after deep brain stimulation surgery

(a) Independent samples t-test					
Variable (T2)	Mean difference (SEM)	Mean T2 (SD).		Effect size (Cohen’s d)	P value*
		Male	Female		
PDSI	9.12 (3.80)	25.16 (12.78)	34.28 (14.66)	0.75	**0.02**
Pain	1.40 (0.74)	4.36 (2.42)	5.76 (3.03)	0.53	0.064
Cognition	0.14 (0.90)	4.57 (3.25)	4.71 (2.93)	0.04	0.88

(b) Mann-Whitney U test for independent samples						
Variable	U-value	Median T2† (IQR)		Z-test	Effect size (Cohen’s d)	P value*
		Male	Female			
GAD-7	532.5	1.5 (4)	7 (7)	2.608	0.33	**0.009**
PHQ-9	478.5	6.5 (6)	8 (11)	1.686	0.22	0.092
Mobility	597	13 (13)	25 (16)	3.592	0.46	**<0.001**
ADL	422.5	8 (8)	11 (9)	0.782	0.10	0.434
Stigma	454.5	4 (5)	6 (8)	1.303	0.17	0.192
Social support	400	1 (3)	2 (4)	0.431	0.06	0.667
Communication	430.5	3 (3)	5 (5)	0.916	0.12	0.36

At 6 months post-operatively, females reported worse overall quality of life, anxiety, depression, and mobility scores than males.

Domains of PDQ-39 Questionnaire tested= Pain, cognition, mobility, ADL, stigma, social support, communication.

* Two-sided p value, alpha level=0.05 (values reaching alpha level in bold face).

† Median T2 values are provided when data were non-normally distributed.

ADL, activities of daily living; F, female; GAD-7, Generalised Anxiety Disorder-7 item questionnaire; M, male; PDSI, Parkinson’s Disease Questionnaire=39 items Summary Index (overall score measure); PHQ-9, Patient Health Questionnaire-9 items (for depression); SEM, Standard error of mean difference; T2, 6 months postoperatively.

**Figure 3:**
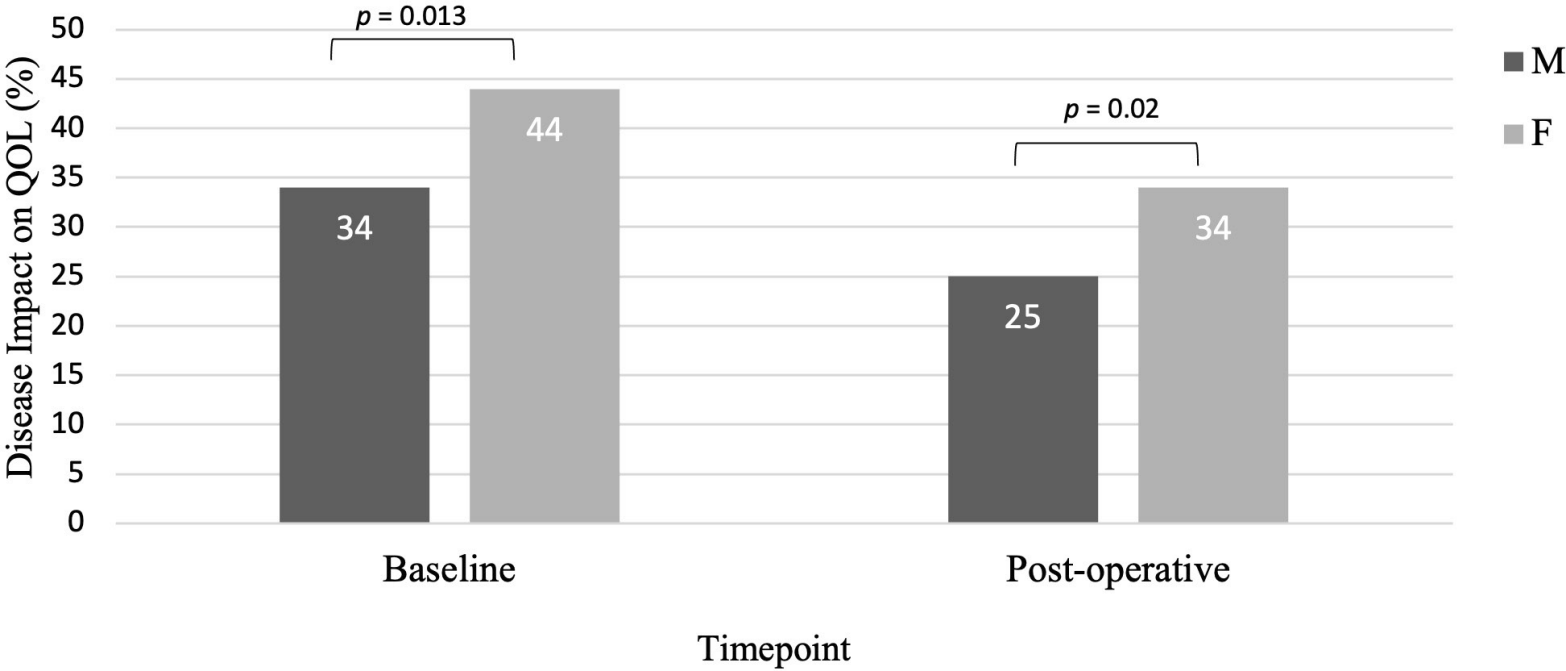
The proportion of change in overall quality of life (QOL) was similar between genders from baseline to 6 months post-operatively, hence females (F) report worse QOL throughout the study compared with males (M). Disease impact on QOL was measured using PDQ-39 Summary Index (PDSI) score, for overall burden of Parkinson’s disease on QOL with 100% representing maximal disease-related burden. Analysed using independent samples t-test, reporting two-sided p values. PDQ-39= Parkinson’s Disease Questionnaire—39 items.

QOL significantly improved in males and females by similar proportions (7.82% p<0.001 and 9.63% p*=*0.004, respectively) (figure 3). Females reported significantly worse postoperative QOL than males (34.28% compared with 25.16%, p*=*0.02) (online supplemental table 7). There was no significant gender difference in the effect of DBS on raw scores in any questionnaire or PDQ-39 domain, except for social support. Social support was significantly worse at baseline in females, who reported scores representing a lack of social support, as median of 3 compared with 1 (*r*-value=0.28, p=0.03). Social support improvement following DBS was significantly better in females than males (p=0.035), who did not report a substantial lack of social support preoperatively or postoperatively (online supplemental table 6). Of the PDQ-39 domains, females reported significantly worse mobility postoperatively (p<0.001), and higher pain scores which demonstrated a trend towards significance (p*=*0.064).

No gender difference was found in the reduction in LEDD (p=0.958, Cohen’s *d*=0.15) nor in the mean dose reduction in PD medication types, except for a greater reduction in male COMT inhibitors dose (p=0.043, Cohen’s *d=0.363*). At 6 months postoperatively, there was no gender difference in LEDD (p*=*0.267, Cohen’s *d=*0.32) nor the average doses of PD medication types (online supplemental table 3).

### Can gender predict changes in anxiety or depression after DBS?

We found that worse postoperative anxiety was significantly predicted by female gender and worse postoperative social support, with these accounting for 23% of the variance in postoperative anxiety scores (*adjusted R^2^ = 0.23, F_(2,58)_=9.90, p*<0.001) (table 3 *model 1*). Postoperative improvement in depression was not significantly predicted by gender, however it was predicted by shorter PD duration and better baseline mobility, accounting for 15.6% of the variance in depression improvement (*adjusted R^2^=0.156, F_(2,58)_= 6.55, p*=0.003) (table 3 *model 2*). Anxiety and depression at baseline and postoperatively were significantly predicted by overall QOL at those timepoints (online supplemental table 8 *model 3*); gender was a non-significant predictor.

**Table 3:** Multivariate linear regression models to identify predictors of effect of deep brain stimulation (DBS) on anxiety (model 1) and depression (model 2)

Variable		B^†^	SE	Beta (β)	T	P value*
Independent	Dependent					
Model 1: GAD-7	(Constant)	1.527	0.621		2.458	0.017
	Gender	2.36	0.959	0.28	2.46	0.017
	Social support (T2)	0.665	0.189	0.399	3.51	<0.001
Model 2: PHQ-9	(Constant)	2.95	2.082		1.417	0.162
	PD duration	−0.582	0.189	−0.383	−3.076	0.003
	Baseline mobility	0.211	0.077	0.343	2.754	0.008

These multivariate linear regression models identify predictors for effect of DBS. Model 1, using the dependent variable of postoperative anxiety, identifies female gender and post-operative social support to explain 23% of the variance in post-operative anxiety scores. Model 2, using the dependent variable of change in PHQ-9 score, identifies disease duration and baseline mobility to account for 15.6% of the variance in depression improvement.

* Two-sided p value, alpha level=0.05.

† B coefficient

GAD-7, Generalised Anxiety Disorder-7 items questionnaire; PD, Parkinson’s disease; PDSI, Parkinson’s Disease Questionnaire-39 Items Summary Index Score (for QOL); PHQ-9, Patient Health Questionnaire-9 items (for depression); T2, 6 months postoperative.

## Discussion

### Depression, anxiety, and QOL improve after DBS surgery across genders

While DBS surgery is approved for routine use in PD for motor symptoms, its impact on non-motor symptoms of PD is less well-defined. We showed DBS to improve non-motor symptoms, finding a reduction in depression and a significant improvement in anxiety and QOL, including within the domains of mobility, pain, and stigma. We identified that improvement in mobility was significantly correlated with improvement in depression. Emerging models designed to predict anxiety improvement after DBS, such as the nomogram to inform preoperative counselling,^20^ could be improved by these findings.

### Females report worse postoperative outcomes for QOL, depression, and anxiety

Our preoperative data indicate that distinct non-motor profiles exist between genders.^6^ Baseline differences showed significantly worse QOL in females, including within pain and mobility domains, consistent with previous studies.^6^ Although baseline clinical cases of anxiety and depression were equal, anxiety scores were higher in females.

Previous studies report mixed outcomes regarding postoperative improvements in either gender.^3 21^ Our postoperative data showed DBS to improve depression, anxiety, and QOL in both genders. We found a greater improvement in clinical depression and anxiety in males, overall challenging the idea of equal clinical efficacy.^6^ Similarly, female gender was a significant predictor of postoperative anxiety.

Of note, these observed results are unlikely to have been contributed to by PD medication changes, given we found no gender difference in postoperative reduction in LEDD nor dopamine agonist dose. In the context of a non-significant difference in LEDD, the only observered gender difference of COMT-inhibitor dose is less likely to be relevant.

### Fewer females undergo DBS than males

Females were under-represented within our study and made up 28% of the cohort, despite comprising 40% of the general PD population.^8^ This replicates several previous studies^6 9 12 22^ and provides evidence that females are less likely to undergo DBS. Females are disproportionately reduced in cohorts at all preoperative stages; from referral, to approved for DBS, to undergoing surgery. Thus the total relative risk of undergoing DBS for females compared with males with PD is 0.74.^6^

One proposed reason for the gender gap is higher rates of depression^6^ in females reducing clinician-approval rates; however our study found no preoperative gender difference in depression. Similarly, other studies have found no gender difference in clinical eligibility^23^ and a significantly higher proportion of approval of females than males for surgery after referral (80% *vs* 69% respectively).^6^ A further reason is that significantly more females decline DBS due to a reduced personal preference for undergoing the procedure.^12^ Our study found females to have more preoperative anxiety and decreased social support, which, alongside the challenges some experience secondary to caregiver roles,^10^ may contribute to their reduced healthcare-seeking behaviours and preference for DBS.^6 12^ Further investigation into the reasons for this gender gap is key to implement gender-sensitive initiatives to address this and help clinicians tailor pre-DBS discussions.

### QOL, mobility, and duration of disease predict effect of DBS on depression

While gender was a non-significant predictor for depression, our models identified relationships with other covariates. Shorter PD duration and better baseline mobility were significant predictors for greater improvement in depression. Given disease duration, motor function, and depression have been correlated with extent of neuronal degeneration^24 25^; this could be driving responsiveness to DBS. Similarly, motor symptoms in advanced PD such as axial instability are less effectively treated by DBS.^26^ While disease duration was similar between genders, the age at clinical presentation and diagnosis may not reflect disease onset since motor symptoms tend to present later in females.^24^ Some data show females undergoing DBS have a longer disease duration and worse mobility at baseline,^6^ which could result in a poorer clinical response, and thus explain the greater symptom burden and poorer QOL in females postoperatively. A limitation to our study is that any life events that may have occurred around the time of DBS surgery and subsequently affected QOL or mental health have not been enquired about or accounted for. Overall, our findings advocate for earlier DBS intervention to improve postoperative outcomes. Early DBS is also supported by expert clinicians^26^, the EARLYSTIM trial demonstrating DBS efficacy over medication for early motor symptoms^27^, and the reduced surgical risk profile earlier in disease.^28^

### Limitations

Our study limitations include the sample size, particularly of females. Reasons for this include the recruitment period spanning during and after the COVID-19 pandemic when most DBS centres reduced the number of procedures done, and initially only five out of seven DBS centres were ready to begin recruitment due to bureaucratic delays.

In addition, the study cohort comprised of a large majority of participants identifying as white British ethnicity (96.7%). All participants were fluent in English hence there were no exclusions based on this recruitment criterion. Instead, this finding reflects a further important inequality in the access to DBS for patients from ethnic minority groups, replicated in previous studies.^29^

Furthermore, while the scale and cut-off score of PHQ-9 is validated,^17^ there remain gaps in the literature for the optimal scale for measuring anxiety in patients with PD. Also, while the use of patient-reported questionnaires reduces the risk of clinician bias, we cannot control for reporting bias. A further limitation is that medication changes were not included as a covariate, however as discussed, given no gender difference was found, this variable is unlikely to contribute to the observed results.

## Conclusion

This study informs on gender differences in DBS cohorts of PD patients, and predictors of postoperative depression and anxiety. DBS improved depression, anxiety, and QOL in both genders. However, there was a greater improvement in males, warranting further research into the biopsychosocial reasons underpinning these gender differences. Both females and patients from ethnic minority groups were under-represented in this DBS cohort, replicating several previous studies. This prompts investigation into the inequities in access to DBS to inform future healthcare strategies, which the Parkinson’s Foundation US created as a national agenda of importance.^7^ We identified potential preoperative barriers to females with PD undergoing DBS to include increased anxiety, reduced social support, and less carer engagement. Improving clinician education of these differences in order to tailor DBS counselling could be important initial steps towards closing the gender gap in DBS.

## Supplementary material

10.1136/bmjno-2025-001246 online supplemental table 1

## Data Availability

Data may be obtained from a third party and are not publicly available. All data relevant to the study are included in the article or uploaded as supplementary information.
