# Supplementary material for: Gender differences in depression, anxiety, and quality of life in Parkinson’s disease before and after deep brain stimulation surgery: a multicentre cohort study
Source: BMJ Neurol Open. 2025 Sep 30;7(2):e001246. doi: 10.1136/bmjno-2025-001246 (PMC12496053; doi:10.1136/bmjno-2025-001246)
Supplement: online supplemental table 1 [file bmjno-7-2-s001.docx]

**SUPPLEMENTARY MATERIAL**

| **Supplementary Table 1**  Participating DBS Centres for CRISP Study | |  |
| --- | --- | --- |
| DBS Centre | City | |
| King's College Hospital | London | |
| Salford Royal NHS | Manchester | |
| NHS Greater Glasgow & Clyde | Glasgow | |
| The Walton Centre NHS Foundation Trust | Liverpool | |
| The Newcastle Upon Tyne Hospitals NHS Foundation Trust | Newcastle | |
| Oxford University Hospitals NHS Foundation Trust | Oxford | |
| Barking, Havering, Redbridge University Hospitals NHS Trust | Romford | |

| Supplementary Table 2  Variables Used in Cohort Study |  |
| --- | --- |
| *Variables* | Type of variables |
| Demographics | |
| Age-at-operation | Continuous |
| Gender | Binary |
| PD duration | Continuous |
| Age-at-PD-diagnosis | Continuous |
| MDS-UPDRS score (part I, II, III, IV) | Continuous |
| Ethnicity | Categorical |
| Recruitment time (pre- vs post-operation) | Binary |
| Carer total number | Continuous |
| Medications (summative doses) |  |
| Levodopa Equivalent Daily Dose (LEDD; calculated using Parkinsonsmeasurement.org) | Continuous |
| Levodopa | Continuous |
| Dopamine agonists (DA) | Continuous |
| MAO-B inhibitors (MAOBI) | Continuous |
| COMT inhibitors (COMTI) | Continuous |
| Anticholinergic | Continuous |
| Psychotropic | Continuous |
| Depression (PHQ-9) | |
| PHQ-9 Total score | Continuous |
| Clinically significant depression Cutoff ≥9 | Binary |
| Anxiety (GAD-7) | |
| GAD-7 | Continuous |
| Clinically significant anxiety Cutoff ≥ 10 | Binary |
| Measures of Quality of Life (PDQ-39 – Self-rated) | |
| PDQ-39 summary index (PDSI) | Continuous |
| Mobility (1-10) | Continuous |
| Activities of Daily Life (11-16) | Continuous |
| Stigma (23-26) | Continuous |
| Social support (27-29) | Continuous |
| Cognition (30-33) | Continuous |
| Communication (34-36) | Continuous |
| Bodily Pain (37-39) | Continuous |

| Supplementary Table 3  Cohort and gender differences in Parkinson’s Disease (PD) medication doses pre- and post-operatively, and reduction following Deep Brain Stimulation surgery (DBS).   \| (a)  *Cohort reduction in PD medication doses following DBS* \| \| \| \| \| \| \| --- \| --- \| --- \| --- \| --- \| --- \| \| Medication type \| Mean  (T0-T2) \| S.D. \| S.E. of mean \| Effect size  (Cohen's *d)* \| *P* value* \| \| LEDD \| 376.639 \| 431.235 \| 55.214 \| 0.736 \| <.001 \| \| Levodopa \| 182.705 \| 256.954 \| 32.9 \| 0.67 \| <.001 \| \| DA \| 11.056 \| 33.041 \| 4.23 \| 0.453 \| 0.011 \| \| MAOBI \| 2.684 \| 14.24 \| 1.823 \| 0.124 \| 0.146 \| \| COMTI \| 80.328 \| 304.724 \| 39.016 \| 0.273 \| 0.044 \| \| Anticholinergic \| 0.098 \| 0.768 \| 0.098 \| 0.165 \| 0.321 \| \| Psychotropic \| -29.574 \| 177.349 \| 22.707 \| -0.15 \| 0.198 \|   *(b)*  *Baseline gender difference in PD medication doses (T0)* | | | | | | |
| --- | --- | --- | --- | --- | --- | --- | --- | --- | --- | --- | --- | --- | --- | --- | --- | --- | --- | --- | --- | --- | --- | --- | --- | --- | --- | --- | --- | --- | --- | --- | --- | --- | --- | --- | --- | --- | --- | --- | --- | --- | --- | --- | --- | --- | --- | --- | --- | --- | --- | --- | --- | --- | --- | --- | --- | --- | --- | --- | --- | --- |
| Medication type | Mean (T0)  Male Female | | Mean Gender Difference (S.E.) | Effect Size (Cohen's *d*) | *P* value* |  |
| LEDD | 1175.93 | 1049.53 | 126.40 (159.95) | 0.223 | 0.433 |  |
| Levodopa | 752.27 | 676.47 | 75.80 (82.11) | 0.26 | 0.36 |  |
| DA | 12.99 | 17.13 | -4.14 (9.48) | -0.123 | 0.664 |  |
| MAOBI | 4.04 | 12.12 | -8.08 (8.41) | -0.357 | 0.349 |  |
| COMTI | 156.82 | 11.76 | 145.05 (59.87) | 0.424 | **0.02** |  |
| Anticholinergic | 0.14 | 0.06 | 0.08 (0.22) | 0.098 | 0.73 |  |
| Psychotropic | 30.18 | 73.06 | -42.88 (35.43) | -0.341 | 0.231 |  |

***(c)***

*Gender difference in reduction in PD medication doses following DBS*

| Medication type | Mean (T0-T2)  Male Female | | Mean Gender Difference (S.E.) | Effect Size  (Cohen's *d*) | | | *P* value* |
| --- | --- | --- | --- | --- | --- | --- | --- |
| LEDD | 378.48 | 371.88 | 6.59 (124.18) | | 0.015 | 0.958 | |
| Levodopa | 177.27 | 196.76 | -19.50(73.95) | | -0.074 | 0.793 | |
| DA | 10.10 | 13.53 | -3.43(9.50) | | -0.102 | 0.719 | |
| MAOBI | 1.43 | 5.94 | -4.52(5.99) | | -0.314 | 0.461 | |
| COMTI | 111.36 | 0.00 | 111.36(53.52) | | 0.363 | **0.043** | |
| Anticholinergic | 0.14 | 0.00 | 0.14 (0.22) | | 0.174 | 0.539 | |
| Psychotropic | -25.93 | -39.00 | 13.07 (51.04) | | 0.072 | 0.799 | |

***(d)***

Gender difference in post-operative medication doses (T2)

| Medication type | Mean (T2)  M F | | Mean Gender Difference (S.E.) | Effect Size (Cohen's *d*) | *P* value* |
| --- | --- | --- | --- | --- | --- |
| LEDD | 797.45 | 677.65 | 119.81 (107.00) | 0.32 | 0.267 |
| Levodopa | 575.00 | 479.71 | 95.29 (69.63) | 0.39 | 0.176 |
| DA | 2.89 | 3.59 | -0.70 (1.42) | -0.14 | 0.622 |
| MAOBI | 2.61 | 6.18 | -3.56 (4.41) | -0.23 | 0.422 |
| COMTI | 45.45 | 11.76 | 33.69 (41.51) | 0.23 | 0.42 |
| Anticholinergic | 0.00 | 0.06 | -0.06 (0.06) | -0.46 | 0.332 |
| Psychotropic | 56.11 | 112.06 | -55.95 (62.63) | -0.25 | 0.375 |
| *Cohort differences were calculated using paired t-tests, and gender differences were calculated using independent samples t-tests, for LEDD and mean dose of each medication type. Overall cohort reduction in PD medications was significant. We found no significant gender difference in LEDD nor mean dose of medication types, except for COMT inhibitors. Males were on a significantly higher COMT inhibitor dose at baseline, thus there was a significantly greater reduction compared to females after DBS.*  *Abbreviations: T0= before DBS. T2= 6-months after DBS. LEDD= Levodopa Equivalent Daily Dose, calculated using ‘www.ParkinsonsMeasurements.Org’. DA= dopamine agonists, MAOBI= Monoamine Oxidase-B Inhibitors, COMTI= Catechol-O-methyl transferase (COMT*) *inhibitors. S.D. = standard deviation*, *S.E=± Standard Error of the difference in means.*  **Two-sided p-value, alpha level=0.05* | | | | | |

| Supplementary Table 4  The Change in Anxiety, Depression, And Quality-Of-Life Scores Following Deep Brain Stimulation Surgery For The Whole Cohort.  (a).  *Paired t-tests* | | | | | | | |  |
| --- | --- | --- | --- | --- | --- | --- | --- | --- |
| Paired differences | *Mean (*±*S.E.)* | | *Cohen’s d Effect size* | | *P value** | |  |  |
| GAD-7 | 2.28 (3.84) | | 0.59 | | **< 0.001** | |  |  |
| PHQ-9 | 1.18 (5.99) | | 0.19 | | 0.13 | |  |  |
| PDSI | 8.32 (11.54) | | 0.71 | | **<.001** | |  |  |
| Mobility | 2.69 (9.01) | | 0.30 | | **0.02** | |  |  |
| ADL | 2.16 (4.33) | | 0.49 | | **<0.001** | |  |  |
| Stigma | 1.39 (3.57) | | 0.39 | | **0.003** | |  |  |
| Cognition | 0.48 (2.85) | | 0.17 | | 0.20 | |  |  |
| Communication | -0.08 (2.75) | | -0.03 | | 0.82 | |  |  |
| (b)  *Wilcoxon signed rank test* | | | | | | | |  |
| Variable | Median difference | r- value effect size | | Z-test statistic | | P-value | | |
| *Social Support* | 1 | -0.14 | | -1.093 | | 0.274 | | |
| *Pain* | 0 | -0.32 | | -2.495 | | **0.013** | | |
| *A significant improvement in anxiety, quality of life, mobility, activities of daily living (ADL) and stigma (Table (a)), as well as in pain scores (Table (b)), was seen from baseline to 6-months after DBS.*  *Abbreviations: GAD-7= Generalised anxiety disorder questionnaire. PHQ-9= Patient health questionnaire for depression. PDSI= Parkinson’s Disease Questionnaire Summary Index (overall score measure). Mobility, Activities of daily living (ADL), stigma, cognition, communication, social support, pain are domains of PDQ-39= Parkinson’s Disease Questionnaire. S.E=± Standard Error of the difference in means.*  **Two-sided p-value, alpha level=0.05* | | | | | | | |  |

| Supplementary Table 5  Baseline gender difference in severity of Parkinson’s disease by MDS-UPDRS scores | | | | | |
| --- | --- | --- | --- | --- | --- |
| MDS-UPDRS | Mean (T0)  Male Female | | Mean Difference | Effect size (Cohen's *d)* | *P value** |
| Part I | 15.34 | 17.12 | -1.777 | -0.284 | 0.318 |
| Part II | 19.48 | 22.41 | -2.934 | -0.373 | 0.191 |
| Part IV | 11.61 | 11.65 | -0.033 | -0.008 | 0.977 |
| *There was no gender difference in UPDRS scores at baseline prior to deep brain stimulation surgery. Note this was not calculated for part III given this data was partially missing and provided for 13 participants only.*  *Abbreviations: Movement Disorder Society-sponsored revision of the Unified Parkinson's Disease Rating Scale (MDS-UPDRS)*  **Two-sided p-value, alpha level=0.05* | | | | | |

| Supplementary Table 6  Gender Differences in Change In Depression, Anxiety And Quality Of Life Domain Scores From Baseline To 6-months After Deep Brain Stimulation (DBS) Surgery  *Independent samples t-test* | | | |
| --- | --- | --- | --- |
| *Variable* | Mean difference (±S.E.) | *Effect size (Cohen’s d)* | *P* value* |
| PHQ-9 | -2.53 (1.69) | -0.42 | 0.14 |
| PDSI | 0.07 (0.76) | 0.15 | 0.57 |
| Social Support | **-1.14 (0.53)** | **-0.61** | **0.035** |
| Pain | -0.67 (0.72) | -0.26 | 0.36 |
| ADL | -0.51 (1.24) | -0.12 | 0.69 |
| Mobility | 1.44 (2.59) | 0.16 | 0.58 |
| Communication | 0.11 (0.79) | 0.04 | 0.89 |
| Cognition | 0.16 (0.82) | 0.05 | 0.85 |
| Stigma | 0.27 (1.17) | 0.07 | 0.82 |

| (b)  *Independent samples Mann Whitney U test* | | | | | | |
| --- | --- | --- | --- | --- | --- | --- |
| Variable (T2-T0) | U-value | Median difference (IQR) | Z-test | Effect size  ( *r-*value) | *P* value |  |
| *GAD-7* | 349.5 | 2 (5) | -0.396 | -0.05 | 0.692 |  |
| *Abbreviations: S.E.= Standard Error of Mean difference IQR= interquartile range. GAD-7= Generalised Anxiety Disorder questionnaire. PHQ-9=Patient Health Questionnaire for depression. PDQ-3=9 Parkinson’s Disease Questionnaire-39 items, PDSI= PDQ-39 Summary Index.*  **Two-sided p value, alpha level=0.05.* | | | | | | |

| Supplementary Table 7  The Gender Difference in Overall Quality Of Life Scores Before And After Deep Brain Stimulation (DBS) Surgery | | | |
| --- | --- | --- | --- |
| *PDSI* | *Mean scores (%) (*±*S.D.)* | | *Gender difference* |
|  | Male | Female |  |
| Baseline | 33.98 (15.46) | 43.91 (13.78) | 9.93% (*p = .01)* |
| Post-operative | 25.16 (12.78) | 34.28 (14.66) | 9.12% (*p = .02)* |
| Difference | 7.82 (11.54, *p* < 0.001) | 9.63 (11.78, *p* = 0.004) |  |
| *The difference in change in scores was similar across genders, hence baseline and post-operative scores for QOL in females remained significantly worse than males, when measured using paired t-tests.*  *Abbreviations: Difference = (Baseline – post-operative). PDSI = Parkinson’s Disease Questionnaire Summary Index, with 100% representing most severe impact on QOL. S.D.= standard deviation, alpha level=0.05, two-sided p values.* | | | |

| Supplementary Table 8  Quality of life predicts anxiety and depression before and 6-months after Deep Brain Stimulation surgery | | | | | | | | | | | |  |
| --- | --- | --- | --- | --- | --- | --- | --- | --- | --- | --- | --- | --- |
| Model 3 | Variables | | | F | | | df | | Adjusted R^2^ | *P* value* | | |
|  | Dependent | | Independent |  |  |  |  |  |  |  |  |  |
| (a) Baseline | GAD-7 | PDSI | | | 10.31 | 1,59 | | 0.134 | | | 0.002 |  |
|  | PHQ-9 | PDSI | | | 23.669 | 1,59 | | 0.274 | | | <0.001 |  |
| (b) Post-operative | GAD-7 | PDSI | | | 15.331 | 1,59 | | 0.193 | | | <0.001 |  |
|  | PHQ-9 | PDSI | | | 69.924 | 1,59 | | 0.535 | | | <0.001 |  |
| *Linear regression model (ANOVA) for pre-operative (a) and 6-months post-operative(b) anxiety (GAD-7) and depression (PHQ-9) scores, showing them to be significantly predicted by overall quality of life.*  *Abbreviations: PDSI = Parkinson’s Disease Questionnaire-39 Summary Index. GAD-7= Generalised anxiety disorder questionnaire. PHQ-9= Patient health questionnaire-9 items for depression.*  **Two-sided p value, alpha level=0.05* | | | | | | | | | | | |  |
